# US practice adoption of patient-engagement strategies and spending for adults with diabetes and cardiovascular disease

**DOI:** 10.1093/haschl/qxad021

**Published:** 2023-06-20

**Authors:** Hector P Rodriguez, Karl Rubio, Chris Miller-Rosales, Andrew J Wood

**Affiliations:** Division of Health Policy and Management, School of Public Health, University of California, Berkeley, 2121 Berkeley Way West #5427, Berkeley, CA 94704, United States; Division of Health Policy and Management, School of Public Health, University of California, Berkeley, 2121 Berkeley Way West #5427, Berkeley, CA 94704, United States; Division of Health Policy and Management, School of Public Health, University of California, Berkeley, 2121 Berkeley Way West #5427, Berkeley, CA 94704, United States; Department of Health Care Policy, Harvard Medical School, 180 Longwood Ave, Boston, MA 02115, United States; Dartmouth Institute for Health Policy and Clinical Practice, Dartmouth College, 1 Medical Center Dr, Lebanon, NH 03756, United States

**Keywords:** patient engagement, diabetes, spending, organization of care, shared decision-making

## Abstract

Patient-engagement strategies are being encouraged by payers and governments, but with limited evidence about whether practice adoption of these strategies impacts utilization and spending. We examine the association of physician practice adoption of patient-engagement strategies (low vs moderate vs high) with potentially preventable utilization and total spending for patients with type 2 diabetes and/or cardiovascular disease using US physician practice survey (n = 2086) and Medicare fee-for-service (n = 736 269) data. In adjusted analyses, there were no differences in potentially preventable utilization associated with practice adoption of patient-engagement strategies. Compared with patients attributed to practices with moderate adoption, patients attributed to practices with high adoption had higher total spending ($26 364 vs $25 991; *P* < .05) driven by spending for long-term services and supports, including home health agency, long-term care, skilled nursing facilities, and hospice payments. In contrast, patients attributed to practices with low adoption had higher total spending ($26 481 vs $25 991; *P* < .01) driven by spending for tests and acute care and clinical access spending. The results highlight that stakeholders that encourage the use of patient-engagement strategies should not necessarily expect reduced spending.

## Lay Summary

Improving the engagement of patients with type 2 diabetes and cardiovascular disease (CVD) in their own health and health care can enhance self-management skills and self-efficacy for behavior change, potentially reducing treatment burden. It remains unclear, however, whether US physician practices with more extensive adoption of patient-engagement strategies, including shared decision making, motivational interviewing, and shared medical appointments, have lower potentially preventable utilization and total spending for adults with type 2 diabetes and/or CVD. In a national study of US physician practices and Medicare beneficiaries, we find that practice adoption of patient-engagement strategies is associated with total spending in a nonlinear fashion. Compared with practices with moderate adoption of patient-engagement strategies, practices with high adoption had higher total spending ($25 991 vs $26 364; *P* < .05) driven by spending for long-term services and supports, while practices with low adoption had higher total spending ($25 991 vs $26 481; *P* < .01) driven by tests, acute care, and clinical access spending. The results highlight that key stakeholders encouraging the use of patient-engagement strategies should not necessarily expect reduced spending.

## Teaser

In a national study of US physician practices and Medicare beneficiaries, practice-level adoption of patient-engagement strategies was associated with total spending in a nonlinear fashion. High practice adoption was associated with higher total spending, driven by spending for long-term services and supports. Low practice adoption was associated with higher total spending, driven by spending for tests, acute care, and clinical access spending. The results highlight that key stakeholders encouraging the use of patient-engagement strategies should not necessarily expect reduced spending.

## Introduction

Adults with diabetes and cardiovascular disease (CVD) can experience high treatment burden and decrements to quality of life, issues that tend to dominate clinical discussions.^1–3^ Improving the engagement of patients with diabetes and CVD in their own health and health care can enhance self-management skills and self-efficacy for behavior change, potentially reducing treatment burden.^4,5^ Preference-sensitive treatment decisions for adults with diabetes include insulin use,^6^ CVD risk prevention,^7–9^ and medication intensification^10^; and preference-sensitive treatment decisions for CVD prevention and management include statin use,^10,11^ stroke-prevention options for atrial fibrillation,^12^ and invasive cardiac care.^13^

Patient-engagement strategies include shared decision making (SDM), motivational interviewing, and shared medical appointments (SMAs), and these approaches can improve patient self-efficacy to navigate treatment decisions, improve treatment adherence, and improve patient-centered outcomes of care.^14–16^ SDM is a collaborative effort of clinicians-patients to engage in deliberative discussions about how treatment options complement patients’ values to determine the treatment choice that best reflects those values and preferences.^17^ Decision aids are evidence-driven tools often used in the SDM process that present the likelihood of potential outcomes for each option, and value clarification exercises to elucidate patients’ goals.^18^

Adults with diabetes and/or CVD are high-priority populations for physician practices and health systems using SDM to provide objective information on benefits/harms to help patients clarify their values and make preference-aligned decisions.^18^ Motivational interviewing involves patient-centered prioritization techniques to support patients with goal-setting for behavior change, including managing cardiovascular risk factors such as smoking cessation, diet, and physical activity.^19,20^ SMAs are medical encounters where clinicians simultaneously meet with multiple patients, reinforcing self-management education and medication management as patients learn from the experiences and treatment of their peers.^21^ SMAs can improve self-management of chronic conditions but can be challenging to implement because of complex logistics.^22^

Research evidence about the impact of patient-level use of patient-engagement strategies for adults with diabetes and CVD, including from randomized controlled trials, indicates that using SDM and motivational interviewing techniques can help patients make informed treatment decisions, can reduce the use of high-cost treatments with limited benefit, and improve self-efficacy for behavior change.^5,8,23^ Implementation research, however, highlights that implementing SDM, motivational interviewing, and SMAs require strongs leadership commitment, workflow adaptations, robust health information technology, and sufficient time and organizational support for staff training.^24–28^

Strategies to improve patient engagement are being encouraged by payers and increasingly being adopted by physician practices.^29,30^ Recent research indicates that physician practice adoption of patient-engagement strategies across medical conditions differs by practice ownership; practices owned by hospital and health systems had lower overall adoption of SDM, motivational interviewing, and SMAs compared with all other ownership types, including independent physician practices.^31^ Patient-engagement strategies are not necessarily intended to reduce spending, but rather to better align treatment plans with patients’ values and preferences. Payers and policymakers, however, sometimes expect that investing in patient-engagement strategies will help reduce spending for acute care.^32^

There is mixed evidence about whether the use of patient-engagement strategies like SDM leads to cost savings.^32,33^ No national evidence exists about the extent to which physician practice adoption of patient-engagement strategies for adults with diabetes and/or CVD is associated with potentially preventable utilization and total spending. Patient-engagement strategies require practice infrastructure and staffing to build their capabilities for engaging patients in SDM, motivational interviewing, and SMAs. Supporting patients in making health care decisions that align with their preferences, however, may lead to higher total spending for Medicare beneficiaries due to greater use of long-term services and supports (LTSS), which include home health agency, long-term care, skilled nursing facilities, and hospice payments.^34^

In this study, we advance the evidence by linking a nationally representative survey of nonfederal US primary care physician practices to Medicare claims data from fee-for-service (FFS) beneficiaries to examine the association of practice adoption of 12 patient-engagement strategies for adults with diabetes and/or CVD with potentially preventable emergency department (ED) and hospital utilization^35^ and total spending.^36,37^ Our approach was guided by the multidimensional framework for patient and family engagement by Carman et al.,^4^ which highlights the important role of direct care engagement strategies for improving quality. Based on the emerging evidence about the impact of SDM and motivational interviewing for adults with diabetes and/or CVD,^23,38–40^ we hypothesized that patients attributed to primary care practices with high adoption of patient-engagement strategies will have a lower odds of potentially preventable utilization because patients are better able to manage their health with resources outside of the ED and hospital but will have higher total spending due to greater use of LTSS.

## Data and methods

We analyzed physician practice responses to the 2017/2018 National Survey of Healthcare Organizations and Systems (NSHOS), a nationally representative sample of nonfederal primary care or multispecialty medical practices with 3 or more primary care physicians, as defined by the 2016 IQVIA OneKey database. Stratified-cluster sampling was used to select physician practices operating under different organizational structures.^41^ A knowledgeable key informant at each practice responded, most often the physician chief or practice manager. The NSHOS included content from the National Study of Physician Organizations and new measures of patient-engagement strategies tested using cognitive interviews with health system leaders and physicians. From 2333 total responses (response rate = 47%), we excluded duplicate surveys and those with high item nonresponse, which resulted in an analytic sample of 2190 physician practices.

NSHOS data were linked to 2017 Medicare Part A and Part B FFS claims data using physician taxpayer identification numbers. Beneficiaries with type 2 diabetes and/or CVD were identified using the Centers for Medicare and Medicaid Services (CMS) Hierarchical Condition Category (HCC) coding. For diabetes, codes for Diabetes with Acute Complications (HCC 17), Diabetes with Chronic Complications (HCC 18), and Diabetes without Complication (HCC 19) were included. For CVD, codes for Cardio-Respiratory Failure and Shock (HCC 84), Congestive Heart Failure (HCC 85), Acute Myocardial Infarction (HCC 86), Unstable Angina and Other Acute Ischemic Heart Disease (HCC 87), Angina Pectoris (HCC 88), Specified Heart Arrhythmias (HCC 96), Cerebral Hemorrhage (HCC 99), Ischemic or Unspecified Stroke (HCC 100), and Vascular Disease (HCC 108) were included.

We attributed beneficiaries to practice locations using methods that the CMS uses as part of their Medicare Shared Savings Program, which is a well-documented and widely accepted method.^42^ This method favors assignment of patients to primary care clinicians over specialists and is based on where patients receive the plurality of their primary care. We used the Medicare claims data to determine diagnoses, potentially preventable utilization, and spending. We used the Master Beneficiary Summary File to capture patient demographics and date of death. The claims data included US Census information for zip code–level socioeconomic data.

A total of 795 659 beneficiaries with diagnosed diabetes and/or CVD were attributed to NSHOS practices. Beneficiaries diagnosed with end-stage renal disease were excluded (n = 15 585) because of different insurance coverage and practice specialization considerations. We also excluded beneficiaries who died in 2017 (n = 43 805). The final analytic sample included 736 269 patients attributed to 1 of 2086 physician practices.

### Independent variable

A composite measure based on survey responses assessing practice adoption of 12 patient-engagement strategies was constructed and transformed to a 0–100 scale (internal consistency reliability, α = 0.87).^43^ The 12 items included the following: (1) SMAs for diabetes; (2) SMAs for CVD; (3) motivational interviewing for smoking cessation, (4) weight loss or diet, (5) physical activity, and (6) medication adherence; (7) training physicians and staff in motivational interviewing; (8) use of decision aids for selecting diabetes medications; (9) physicians and staff formally trained in SDM; (10) physicians routinely engage in SDM; (11) routine use of decision aids; and (12) follow-up with patients on decisions after SDM. Given the paucity of evidence about classifying practices based on patient-engagement strategy adoption, our preference for broad categories to ensure the reliable classification of “high” and “low” adopter practices, and because we anticipated nonlinear associations, we examined whether the top and bottom quartiles of the practice adoption distribution compared with the 2 middle quartiles. Accordingly, practices were categorized based on their adoption of patient-engagement strategies as “low” (0–25th percentile; range: 0–2 strategies), “moderate” (26–75th percentile; range: 3–8 strategies), or “high” (>75th percentile; range: 9–12 strategies). Table S1 summarizes information about practice adoption of each of the 12 patient-engagement strategies overall and stratified by the 3 practice adoption categories.

### Study outcomes

The 3 potentially preventable utilization study outcomes are dichotomous measures reflecting the presence or absence of at least 1 of the following: (1) readmission within 30 days of an index hospitalization for any cause,^44^ (2) hospitalization for ambulatory care sensitive conditions (ACSCs),^45^ and (3) nonemergent visit to the ED.^46^

Readmission within 30 days from the date of an index hospitalization for any cause excluded planned readmissions, admitted to prospective payment system (PPS)–exempt cancer hospitals, as well as beneficiary claims without at least 30 days postdischarge enrollment in FFS Medicare, discharged against medical advice, admitted for primary psychiatric diagnoses, admitted for rehabilitation, or admitted for medical treatment of cancer.

To classify hospitalizations for ACSCs, we identified hospitalizations for ACSCs from International Classification of Diseases, 10th Revision, Clinical Modification (ICD-10-CM) codes that were extracted from the principal diagnosis field of each patient using the following Agency for Healthcare Research and Quality (AHRQ) Prevention Quality Indicators: #1 Diabetes Short-Term Complications, #3 Diabetes Long-Term Complications, #5 Chronic Obstructive Pulmonary Disease or Asthma in Older Adults, #7 Hypertension, #8 Heart Failure, #10 Dehydration, #11 Bacterial Pneumonia, #12 Urinary Tract Infection, #14 Uncontrolled Diabetes, #15 Asthma in Younger Adults, and #16 Lower-Extremity Amputation among Patients with Diabetes.^47^

Nonemergent ED visits are defined by the New York University (NYU) Emergency Department Classification algorithm,^46^ which includes probabilities for classifying 47 132 diagnostic codes. These classifications include probabilities for each diagnosis of being a non-emergency (NE) visit and being primary care treatable (PCT) visit. If NE and PCT visits were greater than 50%, we classified the visit as a nonemergent ED visit.

The primary spending outcome measure is total spending.^36,37^ Dollar denominated spending from attributed patients accrues from both practice providers and non–practice providers. To elucidate sources of potential spending differences between physician practices with varying levels of adoption of patient-engagement strategies, we also examine 8 components of spending: imaging, evaluation and management, procedures, tests, facilities, acute care and clinical access, home health agency (an LTSS), and “other” payments. “Other LTSS” included spending for long-term care, skilled nursing facilities, and hospice payments; these LTSS categories were combined due to small sample sizes that resulted in model nonconvergence when examined separately.

### Covariates

Beneficiary control variables included patient age, race/ethnicity, sex, HCC, risk adjustment factor (RAF) score derived using 2017 claims data to account for patient morbidity,^48^ and dual eligibility for Medicare and Medicaid insurance coverage. We controlled for socioeconomic variables, including the median annual household income within each beneficiary's 5-digit zip code and whether the beneficiary resided in a census tract with high poverty (≥20% of residents at or below the 100% poverty level). Practice control variables included practice ownership, which includes categories of medical group, hospital or health care system, Federally Qualified Community Health Center (FQHC), independently owned, or other ownership. We also controlled for practice size (number of physicians), specialty mix (specialist to primary care physician ratio), the percentage of practice revenue from Medicaid, and state fixed effects to account for state policies that might impact the adoption of patient-engagement strategies.

### Analyses

Unadjusted adoption rates for each of the 12 patient-engagement strategies and physician practice characteristics by the 3 levels of patient-engagement strategy adoption (high vs moderate vs low) were compared. We tested for significant differences in the study variables between adoption levels, using chi-square tests for categorical variables and analysis of variance (ANOVA) for continuous variables.

We estimated 3 generalized linear models (GLMs), using logit as link and binomial as family, to estimate the association of physician practice adoption of patient-engagement strategies with the 3 dichotomous study outcomes of potentially preventable utilization. GLMs using log as link and Gaussian as family estimated the association of physician practice adoption of patient-engagement strategies with total spending and each of 8 components of spending.

To address potential selection effects (ie, practices with high adoption of patient-engagement strategies care for larger shares of clinically complex beneficiaries), we used stabilized inverse probability of treatment weights (IPTWs) in the form of average treatment effect in the treated.^49^ We aimed to have absolute standardized differences of the variables’ means between each of the 3 levels (high vs moderate vs low) of physician practice adoption of patient-engagement strategies to be less than 0.15.^50^ The IPTWs were multiplied by survey weights that accounted for differential sampling and nonresponse of practices and then used to estimate the GLM regressions.^41^

We conducted collinearity and model fit diagnostics for multivariable models. We calculated Variance Inflation Factors (VIFs) for each independent variable, with values greater than 2.0 as an indication of collinearity.

### Sensitivity analysis

The measure of physician practice adoption of patient-engagement strategies was specified as a categorical variable in the main analyses because we anticipated nonlinear effects. We assessed the consistency of the results using a linear specification of the composite measure. To do this, we re-estimated all regression models using a linear specification of the composite measure and estimated dose–response functions,^51^ which estimate generalized propensity scores when the main independent variable is not necessarily normally distributed.

## Results

There were statistically significant differences for all practice characteristics by practice adoption levels (“high” vs “moderate” vs “low”) (Table 1). Practices with high adoption had fewer advanced-practice clinicians compared with practices with moderate adoption (5.1 vs 9.7; *P* < .001), but slightly more advanced-practice clinicians than low adoption practices (5.1 vs 4.4; *P* < .001). A greater proportion of high adoption practices were FQHCs compared with moderate adoption (17.1% vs 14.7%; *P* < .001) and low adoption practices (17.1% vs 10.8%; *P* < .001). Differences in the levels of practice adoption of each individual patient-engagement strategy across categories were large in magnitude and statistically significant (*P* < .001) (Table S1). For example, 72.8% of high adoption practices report having clinicians and staff formally trained in SDM, compared to only 37.7% and 15.3% of moderate and low adoption practices, respectively. Similarly, 99.4% of high adoption practices report using motivational interviewing for medication adherence compared to 61.7% and 0.1% of moderate and low adoption practices, respectively.

**Table 1.** Physician practice characteristics, by practice adoption of patient-engagement strategies (low vs moderate vs high adoption).

	Overall	Low patient-engagement strategies	Moderate patient-engagement strategies	High patient-engagement strategies	Differences		
					Low–moderate	Low–high	Moderate–high
n	2086	532	1037	517			
Practice size, %							
<3 physicians	4.0	4.6	4.2	2.7	***	***	***
3–7 physicians	38.2	39.5	38.8	35.0	**	***	***
8–12 physicians	23.9	25.1	23.7	22.8	***	***	***
13–19 physicians	10.4	10.3	8.7	14.5	***	***	***
20+ physicians	23.5	20.6	24.6	25.0	***	***	
Specialty mix, %							
100% PCPs	19.1	18.4	20.2	17.4	***	***	***
33%–99% PCPs	70.8	70.3	69.0	75.4	***	***	***
<33% PCPs	10.2	11.3	10.8	7.1	**	***	***
Advanced-practice clinician count, mean (SD), %	7.1 (24.5)	4.4 (7.2)	9.7 (34.0)	5.1 (10.3)	***	***	***
Practice ownership, %							
Physician-owned	30.3	28.8	31.9	29.1	***		***
Hospital- or health system–owned	52.9	55.7	53.8	46.8	***	***	***
Other ownership	5.7	3.3	6.7	6.9	***	***	
Federally qualified health center	14.0	10.8	14.7	17.1	***	***	***
Medicaid revenue, %							
No Medicaid revenue	31.7	32.7	30.1	34.1	***	***	***
Low/moderate Medicaid revenue, 1%–29%	55.8	54.4	59.2	50.4	***	***	***
High Medicaid revenue, >30%	12.4	12.9	10.7	15.5	***	***	***
US Census region, %							
Northeast	20.2	21.0	17.9	24.4	***	***	***
Midwest	26.7	23.3	30.1	24.1	***	***	***
South	34.8	39.7	34.0	29.9	***	***	***
West	18.2	16.0	18.1	21.6	***	***	***

Abbreviation: PCP, primary care physician.

**P < 0.01, ***P < 0.001.

Descriptive analyses of attributed Medicare FFS beneficiary characteristics, stratified by practice adoption of patient-engagement strategies (Table 2), indicated substantial differences in patient characteristics by practice adoption level. For example, high adoption practices had higher shares of attributed patients with diabetes and/or CVD who are dually eligible for Medicare and Medicaid and with diagnosed depression compared low and moderate adoption practices. Black and Latino Medicare FFS beneficiaries were more likely to be attributed to practices with low adoption than practices with moderate and high adoption.

**Table 2.** Patient characteristics, by practice adoption of patient-engagement strategies (low vs moderate vs high adoption).

	Overall	Low patient-engagement strategies	Moderate patient-engagement strategies	High patient-engagement strategies	Differences		
					Low–moderate	Low–high	Moderate–high
n	736 269	212 644	355 158	168 467			
Age, mean (SD), y	74.08 (17.85)	74.18 (17.73)	74.14 (16.74)	73.92 (17.38)		***	***
Female, %	53.3	53.4	53	53.4			
Race/ethnicity, %							
Black	8.6	9	8.7	8.1	*	***	***
Hispanic	4.8	5.1	4.7	4.5	**	***	
White	80.4	79.6	81.3	80.5	***	***	***
Other	6.2	6.3	5.3	6.9	***	***	***
Dual Medicare-Medicaid coverage, %	18.5	17.7	17.9	19.9		***	***
Resident of high-poverty neighborhood, %	15.7	16.2	14	16.8	***	**	***
Median household income, mean (SD), $	59 476 (45 668)	59 170 (49 533)	59 731 (40 922)	59 565 (41 201)	***	**	
Hierarchical Condition Category risk factor score, mean (SD)	1.50 (1.90)	1.49 (1.94)	1.50 (1.84)	1.50 (1.78)			
Diabetes, %	58.8	59	59	58.2		**	***
Diabetic retinopathy, %	0.5	0.5	0.5	0.5			
Cardiovascular disease, %	62.6	62.4	62.3	63.1		**	***
Acute myocardial infarction, %	0.9	0.9	0.8	0.9	***		***
Congestive heart failure, %	20.2	20.3	20.3	20			
Coronary artery disease, %	9.8	9.6	9.6	10.2		**	***
Peripheral artery disease, %	21.8	21.6	21.7	22.2		**	**
Stroke and cerebrovascular disease, %	5.8	5.8	5.6	6.1	*	*	***
Cancer, %	10.9	10.9	10.8	11			
Chronic obstructive pulmonary disease, %	14	13.8	14.1	14.1			
Depression, %	7.3	6.7	7.5	7.9	***	***	***

*P < 0.05, **P < 0.01, ***P < 0.001.

In multivariable GLMs estimating each of the 3 measures of potentially preventable utilization, adults with diabetes and/or CVD attributed to physician practices with high adoption of patient-engagement strategies had similar odds of having an all-cause 30-day readmission, hospitalization for ACSCs, and nonemergent ED visits as patients attributed to moderate or low adoption practices (Table 3). Absolute standardized differences of the variables’ means were all found to be less than 0.15 in weighted analyses (Figure S1). Adjusted comparisons of potentially preventable utilization by physician practice adoption levels of patient-engagement strategies are summarized in the Figure S2.

**Table 3.** Generalized linear model results: association of practice adoption of patient-engagement strategies and utilization.

	Adjusted odds ratios		
	Model 1: All-cause 30-day readmission	Model 2: Hospitalization for ambulatory care sensitive condition	Model 3: Unnecessary emergency department visit
n	732 699	732 699	732 699
Practice adoption of patient-engagement strategies			
Low	0.964	0.987	0.981
Moderate (reference)	—	—	—
High	0.991	0.986	0.98
Practice characteristics			
Practice ownership			
Independent (reference)	—	—	—
Physician-owned	1.01	0.959	1.004
Hospital- or health system–owned	1.041	0.951	1.110***
Other ownership	1.075	1.04	1.196***
Federally Qualified Health Center	0.99	1.056	0.971
Practice size			
<3 physicians	1.055	0.948	0.994
3–7 physicians (reference)	—	—	—
8–12 physicians	0.988	0.933**	0.969*
13–19 physicians	1.043	1.007	0.947**
20+ physicians	0.972	0.959	0.950**
Specialty mix			
<33% PCPs	1.04	0.965	1.035
33%–99% PCPs	—	—	—
100% PCPs	1.018	0.96	0.941***
Medicaid revenue			
None	1.005	1.002	1.004
Moderate revenue (1%–29%) (reference)	—	—	—
High revenue, >30%	1.072	1.081*	1.073***
Patient characteristics			
Age (standardized)	1.051***	1.183***	0.977***
Female	1.163***	1.279***	1.336***
Race/ethnicity			
White (reference)	—	—	—
Black	0.859**	1.087*	1.335***
Hispanic	0.933	0.941	1.108***
Other	0.820**	0.839**	0.759***
Dual Medicare-Medicaid coverage	1.135***	1.191***	1.630***
Resident of high-poverty neighborhood	1.058	1.046	1.083***
Hierarchical Condition Category risk factor score	2.106***	2.224***	1.313***

Abbreviation: PCP, primary care physician

The table displays the results of 3 separate adjusted models estimating the association between practice adoption of patient-engagement strategies, practice characteristics, patient characteristics, and (1) all-cause 30-day readmission, (2) hospitalization for ambulatory care–sensitive condition, and (3) unnecessary emergency department visits. State fixed effects were also included in all models. *P < 0.05, **P < 0.01, ***P < 0.001.

Among practice covariates, patients attributed to practices with high Medicaid revenue (>30%) were more likely to have a hospitalization for ACSCs and a nonemergent ED visit than beneficiaries attributed to practices with moderate Medicaid revenue (1%–29%). Patients attributed to practices owned by a hospital or health care systems or “other” had higher rates of nonemergent ED visits. Patients attributed to practices with 100% primary care physicians had lower odds of nonemergent ED visits. All patient covariates included were significantly associated with potentially preventable utilization, but with inconsistent relationship directions across the study outcomes. For example, older beneficiaries had higher all-cause 30-day readmission rates and hospitalization for ACSCs, but lower unnecessary ED visits. Black and Hispanic beneficiaries had higher nonemergent ED visits.

In adjusted GLMs for spending (Table 4), patients attributed to practices with high adoption of patient-engagement strategies had higher total spending (*P* < .05), driven by home health agency and other payments, which include long-term care, skilled nursing facilities, and hospice payments, compared with patients attributed to practices with moderate adoption. In adjusted analyses, patients attributed to practices with low adoption of patient-engagement strategies had higher total spending (*P* < .001), driven by tests, acute care and clinical access, long-term care, skilled nursing facilities, and hospice payments, compared with patients attributed to practices with moderate adoption. Compared with beneficiaries attributed to independent practices, patients attributed to FQHCs had lower total spending (*P* < .001). Most patient characteristics assessed were associated with spending. For example, Black, Hispanic, and beneficiaries with “other” race/ethnicities had lower total spending compared with White beneficiaries, and patients with higher HCC RAF scores had higher total spending.

**Table 4.** Generalized linear model results: association of practice adoption of patient-engagement strategies and spending.

	Total spending	Imaging payments	Evaluation and management payments	Procedures payments	Test payments	Facilities payments	Acute care and clinical access payments	Home health agency	Other payments
n	732 699	732 699	732 699	732 699	732 699	732 699	732 699	732 699	732 699
Practice adoption of patient-engagement strategies									
Low	0.0187**	0.00908	−0.0108*	−0.00937	0.0156**	0.0174	0.0353*	−0.0282	0.0454**
Moderate (reference)	—	—	—	—	—	—	—	—	—
High	0.0142*	0.0038	−0.00637	−0.0103	0.00432	0.0179	0.0266	0.0519*	0.0373*
Practice characteristics									
Practice ownership									
Independent (reference)	—	—	—	—	—	—	—	—	—
Physician-owned	0.0156	0.0599***	0.00763	0.0238	0.0616***	0.0771***	−0.00967	0.0823*	0.0208
Hospital- or health system–owned	0.00251	0.127***	−0.0177**	0.0236*	−0.00495	0.280***	0.0178	0.00549	−0.0456*
Other ownership	−0.0319*	0.328***	−0.194***	−0.0438*	0.0403**	0.140***	−0.0268	0.0836	−0.132***
Federally Qualified Health Center	−0.028***	0.107***	−0.0567***	−0.0572***	−0.043***	−0.10***	−0.0196	−0.0462	−0.098***
Practice size									
<3 physicians	−0.00365	0.0535***	0.000902	−0.00126	−0.085***	−0.00428	−0.0344	−0.0043	0.0579
3–7 physicians (reference)	—	—	—	—	—	—	—	—	—
8–12 physicians	−0.00039	−0.023***	0.0204***	−9.60E-05	−0.022***	−0.135***	−0.0271	0.127***	0.0225
13–19 physicians	0.0427***	0.0102	0.0638***	0.0649***	−0.027***	−0.230***	0.016	0.126***	0.0632*
20+ physicians	0.0250**	0.0259**	0.00778	0.00244	−0.00408	−0.188***	0.0443*	0.0992**	0.0302
Specialty mix									
<33% PCPs	0.0207	0.0646***	−0.0166	0.0132	0.0339***	0.206***	0.0229	0.0258	0.00343
33%–99% PCPs	—	—	—	—	—	—	—	—	—
100% PCPs	0.0367***	−0.033***	0.0662***	0.0434***	0.0492***	0.0318	0.0750***	0.00638	0.00159
Medicaid revenue									
None	−0.0024	0.0579***	0.0107*	0.0151	0.0539***	−0.081***	−0.00544	−0.074***	−0.0464**
Moderate revenue (1%–29%) (reference)	—	—	—	—	—	—	—	—	—
High revenue, > 30%	−0.0125	0.115***	−0.0916***	−0.0454***	−0.030***	0.203***	−0.000278	0.0666	−0.0432
Patient characteristics									
Age	−0.023***	−0.112***	−0.0544***	−0.0855***	−0.116***	−0.104***	−0.127***	0.432***	0.162***
Female	0.0768***	0.110***	0.0944***	−0.0917***	0.0212***	0.707***	0.00867	0.424***	0.296***
Race/ethnicity									
White (reference)	—	—	—	—	—	—	—	—	—
Black	−0.173***	−0.096***	−0.0943***	−0.294***	−0.102***	−0.081***	−0.289***	0.182***	−0.215***
Hispanic	−0.162***	−0.0434**	−0.131***	−0.188***	−0.147***	−0.0506	−0.143***	−0.0966	−0.256***
Other	−0.147***	−0.107***	−0.112***	−0.179***	−0.122***	−0.196***	−0.145***	−0.225***	−0.195***
Dual Medicare-Medicaid coverage	−0.137***	−0.206***	−0.0499***	−0.246***	−0.132***	−0.072***	−0.373***	0.495***	−0.0358
Resident of high-poverty neighborhood	−0.0189*	−0.00404	−0.0315***	−0.0672***	−0.0206**	0.0017	0.00603	0.0312	−0.0348
Hierarchical Condition Category risk factor score	0.746***	0.379***	0.552***	0.371***	0.312***	0.209***	1.132***	1.009***	0.897***

Abbreviation: PCP, primary care provider.

The table displays the results of 9 separate adjusted models estimating the association between practice adoption of patient-engagement strategies, practice characteristics, patient characteristics, and number. State fixed effects were also included in all models. *P < 0.05, **P < 0.01, ***P < 0.001.

**Figure 1.**
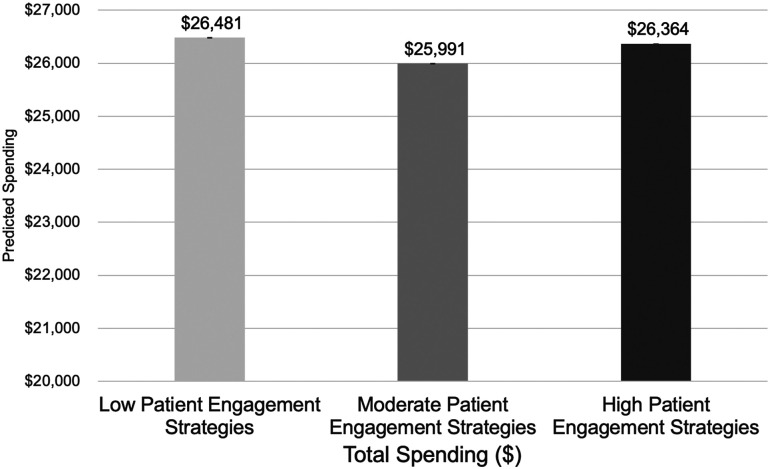
Predicted values for total annual (2017) spending for Medicare fee-for-service beneficiaries, by practice adoption of patient-engagement strategies.

Predicted mean estimates of adjusted total spending are illustrated in the Figure 1 and show that Medicare FFS beneficiaries with diabetes and/or CVD attributed to physician practices with “moderate” adoption of patient-engagement strategies had lower total spending than beneficiaries attributed to practices with low adoption ($25 991 vs $26 481; *P* < .01) or high adoption ($25 991 vs $26 364; *P* < .05) of patient-engagement strategies.

In sensitivity analyses that estimated the association of a continuous specification of the practice adoption of patient-engagement strategies measures, there was no significant association between practice adoption of patient-engagement strategies with preventable utilization (Table S2), consistent with the main analyses. The spending results (Table S3) indicate that a 1-point increase in practice adoption of patient-engagement strategies (range: 0–12) is associated with a $56.7 decrease in total spending per beneficiary (*P* < .001), driven by lower “other payments” (= −$41.2; *P* < .001), which includes spending for long-term care, skilled nursing facilities, and hospice payments, as well as evaluation and management spending (= −$6.7; *P* < .001).

## Discussion

We found practice adoption of patient-engagement strategies to be associated with total spending in a nonlinear fashion. Practices with high adoption of strategies spent more on LTSS for Medicare FFS beneficiaries, including home health agency, skilled nursing facility, hospice, and long-term care payments, compared with practices with low and moderate adoption. Higher spending for LTSS may be indicative of patient-centered care for these clinically complex beneficiaries. The results suggest that achieving patient-centered care may actually increase LTSS spending, while not associated with reduced potentially preventable utilization in the short run.^52^ In contrast, low adoption practices spent more on testing, acute care, and clinical access payments for Medicare FFS beneficiaries than moderate adoption practices. Higher spending for these services may reflect treatment that may not be aligned with patients’ preferences, less developed diabetes and CVD self-management support for patients, differences in geographic access to care, and care provision in critical access hospitals, which tend to be in rural settings. Black and Latino Medicare FFS beneficiaries were more likely to be attributed to physician practices with low adoption of patient-engagement strategies, so addressing implementation barriers impeding practice adoption and implementation of patient-engagement strategies in these practices through technical assistance and other resources to promote patient-centered care may also advance health equity and help control the growth rate of Medicare spending.

In sensitivity analyses that estimated the association of practice adoption as a continuous measure in dose–response models, greater practice adoption of patient-engagement strategies was associated with lower total spending. These results were driven by lower spending for long-term care, skilled nursing facilities, and hospice payments and contrast with our main specification, which found the opposite relationship. While dose–response models are more robust for reliably estimating independent variables that are not necessarily normally distributed, we present the categorical measure of practice adoption as our main specification to illustrate differences more clearly between beneficiaries attributed to practices with high versus low adoption. Taken together, our findings suggest that practice adoption of patient-engagement strategies are generally associated with lower spending for beneficiaries with diabetes and/or CVD, but practices with high adoption may experience diminishing returns to spending reductions due to their efforts to address the complexity of beneficiaries’ clinical and social needs.

Medicare FFS beneficiaries with diabetes and/or CVD attributed to physician practices with high adoption of patient-engagement strategies, however, did not translate into lower odds of having an inpatient readmission for any cause within 30 days, hospitalization for ACSCs, or a nonemergent ED visit. Other practice-level variables were not associated with 30-day readmission or hospitalization for ACSCs. Patients attributed to hospital- or health system–owned practices, smaller practices, and practices with high specialist composition, however, had higher odds of nonemergent ED visits. These results collectively suggest that structural characteristics of physician practices, such as ownership and specialty composition, may affect potentially preventable utilization more than practice-level adoption and use of patient-engagement strategies.

Overall, our results indicate that high practice adoption of patient-engagement strategies may enable the provision of patient-centered care for Medicare FFS beneficiaries with chronic conditions but may not necessarily reduce potentially preventable utilization. There are several reasons for the lack of association of practice adoption on potentially preventable utilization. SDM is often implemented by health care systems and physician practices with low fidelity to evidence-based SDM processes.^53^ As a result, decision-aid use can sometimes be associated with the utilization of high-cost services.^18,54^ High adoption practices all use motivational interviewing support for behavior change, which can require substantial non-clinician staff resources to monitor goals and follow-up on treatment decisions.^55^ SDM and motivational interviewing may also shift older Medicare FFS patients with diabetes and/or CVD to use supportive and community-based services, including home health and long-term care, which can improve their quality of life. More robust organizational support and payment reform may be needed to improve the implementation of patient-engagement strategies so that they reduce preventable utilization for adults with diabetes and/or CVD.

This study has some limitations that should be considered. First, the NSHOS was completed by a single respondent. Respondents were selected for their experience, knowledge, and understanding of organizational processes, but they may have overreported adoption. The modest overall adoption levels are indicative that any social desirability biases are likely small in magnitude. Second, the patient-engagement strategies do not cover all preference-sensitive treatment decisions relevant to patients with diabetes and/or CVD, including decision aids for atrial fibrillation and invasive cardiac care. Third, our results may not generalize to populations not covered by Medicare FFS. Future research should examine other populations when national data are available. Fourth, NSHOS excludes practices with fewer than 3 primary care physicians, so the results may not be generalizable to small practices. Fifth, we did not assess physician-level implementation. Physician- and practice-level variation about the reach, depth, and fidelity of implementation of patient-engagement strategies using multi-informant surveys, interviews, or electronic health record data can clarify the extent to which implementation differences are associated with potentially preventable utilization and spending^56^ and this should be assessed in future research. Finally, although we used propensity score methods to help account for potential selection effects, these methods cannot account for unmeasured factors that might impact the study results.^57^

## Conclusion

US physician practices with high and low adoption of patient-engagement strategies have higher spending for Medicare FFS beneficiaries with diabetes and/or CVD compared with practices with moderate adoption, but the higher spending is for different types of care. Higher spending for practices with high adoption of patient-engagement strategies was driven primarily by home health agency spending, while higher spending for low adoption practices was driven by tests and acute care and clinical access spending. The results reinforce that stakeholders that encourage the use of patient-engagement strategies should not necessarily expect reduced spending.

## Supplementary Material

qxad021_Supplementary_Data
