# Supplementary material for: US practice adoption of patient-engagement strategies and spending for adults with diabetes and cardiovascular disease
Source: Health Aff Sch. 2023 Jun 20;1(1):qxad021. doi: 10.1093/haschl/qxad021 (PMC11103728; doi:10.1093/haschl/qxad021)
Supplement: qxad021_Supplementary_Data [file qxad021_Supplementary_Data.zip › HA_Scholar_Supplemental.docx]

**Supplemental Material**

**U.S. Practice Adoption of Patient Engagement Strategies and Spending for Adults with Diabetes and Cardiovascular Disease**

Table of Contents

**Table 1.** Practice Adoption of Twelve Patient Engagement Strategies, Overall and by Practice Adoption Category

**Figure 1.** Balance between practice adoption categories before and after propensity score weighting

**Figure 2:** Adjusted Utilization Outcomes, by Practice Adoption Category

**Table 2.** Dose-Response Model Results: Association of Practice Adoption of Patient Engagement Strategies and Utilization

**Table 3.** Dose-Response Model Results: Association of Practice Adoption of Patient Engagement Strategies and Spending

**Table 1. Practice Adoption of Twelve Patient Engagement Strategies, by Overall Practice Adoption Level**

|  | **Overall** | **Low Patient Engagement Strategies** | **Moderate Patient Engagement Strategies** | **High Patient Engagement Strategies** | **Difference between groups** | | |
| --- | --- | --- | --- | --- | --- | --- | --- |
| Practice n | 2,086 | 532 | 1,037 | 517 | Low - Moderate | Low - High | Moderate - High |
| 1. Use of motivational interviewing techniques by clinicians and staff | 28.1% | 4.7% | 25.9% | 66.0% | -0.212*** | -0.613*** | -0.401*** |
| 2. Motivational interviewing for smoking cessation | 54.0% | 0.8% | 67.1% | 99.7% | -0.663*** | -0.989*** | -0.326*** |
| 3. Motivational interviewing for weight loss or diet | 54.4% | 0.2% | 68.2% | 99.7% | -0.680*** | -0.995*** | -0.315*** |
| 4. Motivational interviewing for increasing physical activity | 53.1% | 0.0% | 65.8% | 99.5% | -0.658*** | -0.995*** | -0.337*** |
| 5. Motivational interviewing for medication adherence | 51.1% | 0.1% | 61.7% | 99.4% | -0.616*** | -0.993*** | -0.377*** |
| 6. Clinicians and staff formally trained in shared decision-making | 38.4% | 15.3% | 37.7% | 72.8% | -0.224*** | -0.575*** | -0.351*** |
| 7. Routinely engage in shared decision-making | 51.0% | 31.1% | 52.3% | 76.2% | -0.212*** | -0.451*** | -0.239*** |
| 8. Routinely use decision aids | 38.7% | 20.5% | 38.5% | 64.9% | -0.180*** | -0.444*** | -0.264*** |
| 9. Follow-up after shared decision-making | 45.8% | 27.4% | 47.0% | 69.1% | -0.196*** | -0.416*** | -0.221*** |
| 10. Decision aid use for selecting medication for diabetes | 35.1% | 16.7% | 35.5% | 60.3% | -0.188*** | -0.436*** | -0.249*** |
| 11. Shared medical appointments for cardiovascular disease patients | 5.9% | 1.0% | 2.1% | 21.5% | -0.012*** | -0.205*** | -0.193*** |
| 12. Shared medical appointment for patients with diabetes | 10.6% | 2.2% | 6.1% | 32.5% | -0.038*** | -0.302*** | -0.264*** |

Note: ***, **, and * indicate p<0.001, p<0.01, and p<0.05, respectively.

**Figure 1. Balance between physician adoption groups before and after propensity score weighting**

Figure 1a) Figure 1b)


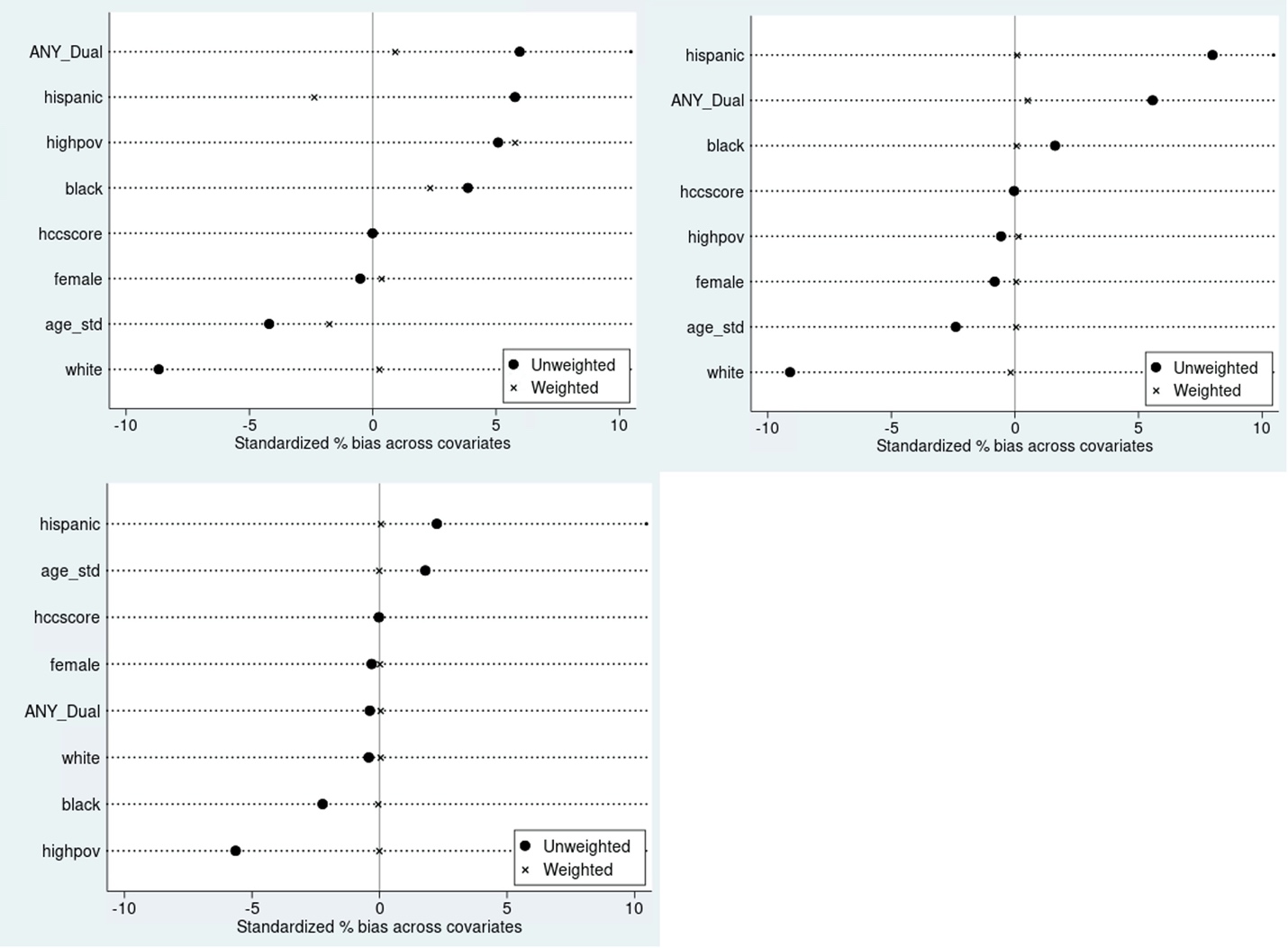


Figure 1c)

Note: (1a) *upper left* balance between practices with low vs. moderate patient engagement strategies adoption, (1b) *upper right* balance between practices with low vs. high patient engagement strategies adoption, (1c) *lower left* balance between practices with moderate vs. high patient engagement strategies adoption

**Figure 2: Adjusted Utilization Outcomes, by Practice Adoption Category**


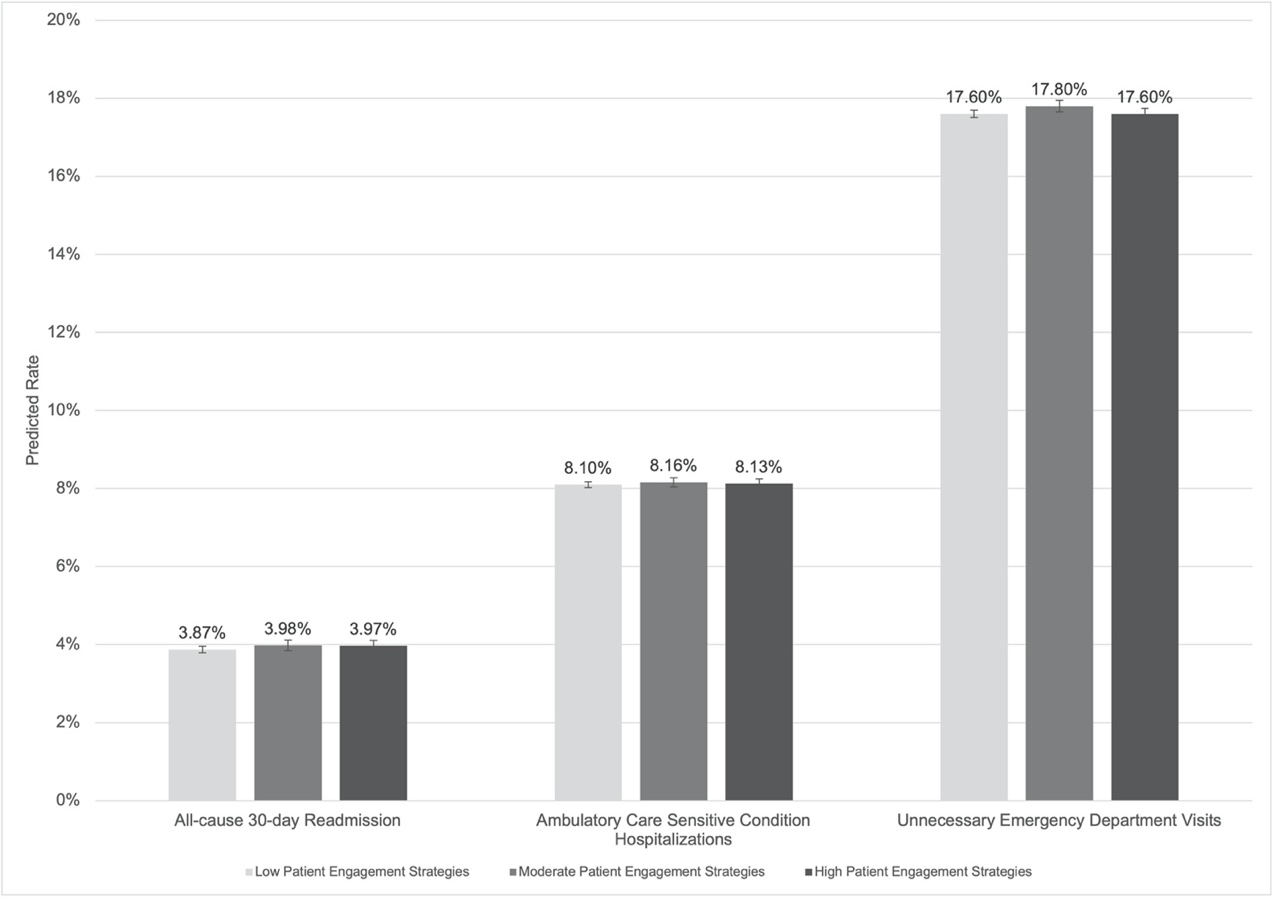


**Table 2. Dose-Response Model Results: Association of Practice Adoption of Patient Engagement Strategies and Utilization**

|  | **Average Treatment Effects (OLS Terms)** | | |
| --- | --- | --- | --- |
|  | **All-cause 30-day Readmission** | **ACSC Hospitalization** | **Unnecessary ED visit** |
| N | 732,699 | 732,699 | 732,699 |
| Patient Engagement Strategy Composite (range: 0-12) | 0.000 | 0.000 | 0.000 |
| **Practice Characteristics** |  |  |  |
| Practice Ownership |  |  |  |
| Independent (reference) | - | - | - |
| Physician-owned | -0.000 | 0.001 | -0.003* |
| Hospital- or health system-owned | 0.001* | 0.002* | 0.014*** |
| Other ownership | 0.003** | 0.005** | 0.026*** |
| Federally Qualified Health Center | -0.002* | 0.001 | -0.005** |
| Practice Size |  |  |  |
| <3 physicians | 0.002 | -0.000 | 0.002 |
| 3-7 physicians (reference) | - | - | - |
| 8-12 physicians | 0.001 | 0.001 | 0.000 |
| 13-19 physicians | 0.001 | 0.001 | -0.005** |
| 20+ physicians | 0.001 | 0.001 | -0.000 |
| Specialty Mix |  |  |  |
| <33% PCP | 0.002* | 0.003** | 0.008*** |
| 33-99% PCP | - | - | - |
| 100% PCP | 0.002** | -0.001 | -0.002 |
| Medicaid Revenue |  |  |  |
| None | 0.000 | 0.001 | 0.003** |
| Moderate revenue (1-29%) (reference) | - | - | - |
| High revenue, >30% | 0.003*** | 0.005*** | 0.010*** |
| **Patient Characteristics** |  |  |  |
| Age (standardized) | -0.005*** | 0.002*** | -0.005*** |
| Female | 0.005*** | 0.013*** | 0.039*** |
| Race/Ethnicity |  |  |  |
| White (reference) | - | - | - |
| Black | -0.005*** | 0.006*** | 0.047*** |
| Hispanic | -0.001 | 0.002 | 0.011*** |
| Other | -0.001 | -0.001 | -0.042*** |
| Dual Medicare-Medicaid coverage | -0.005*** | -0.001 | 0.085*** |
| Resident of high poverty neighborhood | -0.001 | 0.002* | 0.007*** |
| Hierarchical Condition Category Risk Factor Score | 0.051*** | 0.085*** | 0.048*** |

Note: ***, **, and * indicate p<0.001, p<0.01, and p<0.05, respectively.

**Table 3. Dose-Response Model Results: Association of Practice Adoption of Patient Engagement Strategies and Spending**

|  | **Total Spending** | **Imaging Payments** | **Evaluation and Management Payments** | **Procedures Payments** | **Tests Payments** | **Facilities Payments** | **Acute Care and Clinical Access Payments** | **Other Payments** |
| --- | --- | --- | --- | --- | --- | --- | --- | --- |
| N | 732,699 | 732,699 | 732,699 | 732,699 | 732,699 | 732,699 | 732,699 | 732,699 |
| Patient Engagement Strategy Composite (range: 0-12) | -56.7*** | 4.2*** | -6.7*** | -1.1 | -0.8 | -0.0 | 1.6 | -41.2*** |
| **Practice Characteristics** |  |  |  |  |  |  |  |  |
| Practice Ownership |  |  |  |  |  |  |  |  |
| Independent (reference) | - | - | - | - | - | - | - | - |
| Physician-owned | 346.9*** | 31.5*** | 31.7** | 10.1 | 18.0*** | 0.4 | 205.7*** | 42.3 |
| Hospital- or health system-owned | 430.6*** | 109.6*** | -7.7 | 31.6** | -16.8*** | 5.8*** | 407.9*** | -122.1*** |
| Other ownership | 794.1*** | 264.7*** | -142.9*** | -37.1 | -14.7** | 3.8*** | 618.9*** | 13.2 |
| Federally Qualified Health Center | -282.7*** | 89.6*** | -76.4*** | -102.2*** | -31.0*** | -1.1*** | 10.6 | -166.9*** |
| Practice Size |  |  |  |  |  |  |  |  |
| <3 physicians | 152.2 | 19.9* | 10.6 | 41.4 | -56.0 | 3.0*** | -124.5 | 303.1*** |
| 3-7 physicians (reference) | - | - | - | - | - | - | - | - |
| 8-12 physicians | 224.5*** | 14.0*** | 41.2*** | -2.0 | 9.7*** | -0.9*** | 32.3 | 143.6*** |
| 13-19 physicians | 520.7*** | 31.6*** | 70.9*** | 60.7*** | 9.6*** | -1.1*** | 213.8*** | 78.4*** |
| 20+ physicians | 426.1*** | 62.8*** | 23.7* | 25.2* | 13.7*** | -1.0*** | 367.0*** | -30.5 |
| Specialty Mix |  |  |  |  |  |  |  |  |
| <33% PCP | -19.1 | 14.1** | -20.1 | -3.4 | 1.5 | 1.6*** | 69.7 | -165.0*** |
| 33-99% PCP | - | - | - | - | - | - | - | - |
| 100% PCP | 481.6*** | 2.8 | 108.7*** | 58.0*** | 30.6*** | 0.6*** | 276.8*** | -30.2 |
| Medicaid Revenue |  |  |  |  |  |  |  |  |
| None | 50.5 | 13.4*** | -0.0 | 14.8 | 29.5*** | -1.0*** | 35.6 | -44.5 |
| Moderate revenue (1-29%) (reference) | - | - | - | - | - | - | - | - |
| High revenue, >30% | 87.2 | 95.8*** | -121.4*** | -28.8* | 2.6 | 1.5*** | 127.1** | 36.4 |
| **Patient Characteristics** |  |  |  |  |  |  |  |  |
| Age | -1,793*** | -98.4*** | -215.6*** | -199.3*** | -75.0*** | 2.0*** | -1,341*** | 345.3*** |
| Female | 896.4*** | 70.1*** | 125.3*** | -202.5*** | 14.0*** | 11.7*** | 70.1** | 856.7*** |
| Race/Ethnicity |  |  |  |  |  |  |  |  |
| White (reference) | - | - | - | - | - | - | - | - |
| Black | -1,055*** | -63.4*** | -61.4*** | -383.8*** | -26.4*** | -1.3*** | -436.8*** | 50.7 |
| Hispanic | -715.4*** | -27.2*** | -93.2*** | -290.9*** | -51.7*** | -1.2*** | 110.6 | -241.4*** |
| Other | -204.7 | -15.7* | -78.1*** | -237.8*** | -48.7*** | -3.6*** | 413.3*** | -134.2* |
| Dual Medicare-Medicaid coverage | -4,355*** | -199.2*** | -311.8*** | -576.2*** | -97.6*** | -1.3*** | -2,484*** | -577.5*** |
| Resident of high poverty neighborhood | -176.2** | -4.1 | -50.8*** | -108.6*** | -19.0*** | -0.5*** | 20.0 | -1.3 |
| Hierarchical Condition Category Risk Factor Score | 14,593*** | 363.7*** | 1,293*** | 785.5*** | 205.0*** | 3.6*** | 7,249*** | 3,943*** |

Note: Home health agency payments are included with “other payments” because the dose-response model for this category of spending did not converge. ***, **, and * indicate p<0.001, p<0.01, and p<0.05, respectively.
